# Supplementary material for: Mapping the evolution of fertility support policies in China: A content and instrumental analysis
Source: PLoS One. 2025 Oct 9;20(10):e0332137. doi: 10.1371/journal.pone.0332137 (PMC12510515; doi:10.1371/journal.pone.0332137)
Supplement: S1 Appendix — (ZIP) [file pone.0332137.s001.zip › S1 Appendix. 226 original policy documents/188-国家发展改革委办公厅、国家卫生健康委办公厅关于组织实施普惠托育服务专项行动的通知(FBM-CLI-4-339056).doc]

国家发展改革委办公厅、国家卫生健康委办公厅关于组织实施普惠托育服务专项行动的通知

**发布部门：** [国家发展和改革委员会(含原国家发展计划委员会、原国家计划委员会)](https://www.pkulaw.com/law/chl?Aggs.IssueDepartment=60301) [国家卫生健康委员会](https://www.pkulaw.com/law/chl?Aggs.IssueDepartment=60321) [机构沿革](javascript:void(0))

**发文字号：**发改办社会〔2020〕74号

**发布日期：**2020.01.22

**实施日期：**2020.01.22

**时效性：** [现行有效](https://www.pkulaw.com/law/chl?Aggs.TimelinessDic=01)

**效力级别：** [部门规范性文件](https://www.pkulaw.com/law/chl?Aggs.EffectivenessDic=XE0302)

**法规类别：** [机关工作综合规定](https://www.pkulaw.com/law/chl?Aggs.Category=00301)

国家发展改革委办公厅、国家卫生健康委办公厅关于组织实施普惠托育服务专项行动的通知
（发改办社会〔2020〕74号）

各省、自治区、直辖市及计划单列市、新疆生产建设兵团、黑龙江农垦总局发展改革委、卫生健康委：
　　党中央、国务院高度重视3岁以下婴幼儿托育服务发展。中央经济工作会议部署“要重视解决好‘一老一小’问题”“支持社会力量发展普惠托育服务”的工作要求。《[国务院办公厅关于促进3岁以下婴幼儿照护服务发展的指导意见](https://www.pkulaw.com/chl/434b24b8b5769383bdfb.html)》明确提出，“地方各级政府要将需要独立占地的婴幼儿照护服务设施和场地建设布局纳入相关规划，新建、扩建、改建一批婴幼儿照护服务机构和设施”。为确保工作目标任务落实落地，请按照《支持社会力量发展普惠托育服务专项行动实施方案（试行）》（发改社会〔2019〕1606号），加大组织实施力度，广泛动员各方资源扩大普惠托育服务项目储备，有关事项通知如下。
　　**一、**请各地发展改革委向政府分管领导报告，会同卫生健康、教育等部门集中攻关，基于调查分析提出符合实际的工作目标，明确资金需求，并组织项目申报。各地卫生健康部门要积极主动与发展改革委对接，动员有条件的单位参与项目申报，配合做好项目审核，加强行业综合监管。

　　**二、**原则上，可按以下标准组织项目申报：5000万人口以上省份，要推荐3个及以上城市参与试点，申报托位合计5000个；3000～5000万人口省份，要推荐3个及以上城市参与试点，申报托位合计3000个；3000万人口以下省份，推荐2个及以上城市参与试点，申报托位合计2000个；直辖市申报托位1000个；计划单列市申报托位500个。同时，积极对接并服务于国家重大战略，专项行动投资安排优先向贫困地区、长江经济带、黄河流域等区域符合条件的项目倾斜。

　　**三、**分批推进2020年专项行动。第一批投资计划建议请按《[关于编报普惠托育服务专项行动2020年中央预算内投资项目计划建议的通知](https://www.pkulaw.com/chl/c619928fc4ebc77dbdfb.html)》（发改办社会〔2019〕1090号）要求，抓紧上报。第二批投资计划建议请参照本通知要求，于2月20日前正式报送。
　　联系方式：
　　国家发展改革委社会司：
　　陈敏010-68502380；贺婷 010-68502517
　　卫生健康委规划司
　　蔡晓琴 010-68791229
　　卫生健康委人口家庭司
　　李　瑞 010-62030560；陈晨 010-62030586

国家发展改革委办公厅
国家卫生健康委办公厅
2020年1月22日

© 北大法宝：（[www.pkulaw.com](https://www.pkulaw.com/)) 专业提供法律信息、法学知识和法律软件领域各类解决方案。北大法宝为您提供丰富的参考资料，正式引用法规条文时请与标准文本核对。
欢迎查看所有[产品和服务](https://www.pkulaw.com/helps/2.html)。法宝快讯：[如何快速找到您需要的检索结果？](https://www.pkulaw.com/helps/70.html)[法宝](https://www.pkulaw.com/helps/69.html)V6有何新特色？

扫描二维码阅读原文

**原文链接：**<https://www.pkulaw.com/chl/2d278255bc6f72c4bdfb.html>
